# Compensation claims for chiropractic in Denmark and Norway 2004–2012

**DOI:** 10.1186/s12998-014-0037-4

**Published:** 2014-11-07

**Authors:** Jørgen Jevne, Jan Hartvigsen, Henrik Wulff Christensen

**Affiliations:** Hønefoss Kiropraktikk og Rehabilitering, Torvgata 2, 3513 Hønefoss, Norway; Department of Sports Science and Clinical Biomechanics, Faculty of Health Sciences, University of Southern Denmark, Campusvej 55, DK-5230 Odense M, Denmark; Nordic Institute of Chiropractic and Clinical Biomechanics, Campusvej 55, DK-5230 Odense M, Denmark

**Keywords:** Compensation claims, Chiropractic, Manual therapy, Adverse events, Side effects

## Abstract

**Background:**

Adverse events are commonly observed in all parts of health care and have been reported extensively following manual therapy, including chiropractic. The majority of reported adverse events following chiropractic care are mild, transitory and self-limiting. However, little is known about patient filed compensation claims related to the chiropractic consultation process. The aim of this study was to describe claims reported to the Danish Patient Compensation Association and the Norwegian System of Compensation to Patients related to chiropractic from 2004 to 2012.

**Methods:**

All finalized compensation claims involving chiropractors reported to one of the two associations between 2004 and 2012 were assessed for age, gender, type of complaint, decisions and appeals. Descriptive statistics were used to describe the study population.

**Results:**

338 claims were registered in Denmark and Norway between 2004 and 2012 of which 300 were included in the analysis. 41 (13.7%) were approved for financial compensation. The most frequent complaints were worsening of symptoms following treatment (n = 91, 30.3%), alleged disk herniations (n = 57, 19%) and cases with delayed referral (n = 46, 15.3%). A total financial payment of €2,305,757 (median payment €7,730) were distributed among the forty-one cases with complaints relating to a few cases of cervical artery dissection (n = 11, 5.7%) accounting for 88.7% of the total amount.

**Conclusion:**

Chiropractors in Denmark and Norway received approximately one compensation claim per 100.000 consultations. The approval rate was low across the majority of complaint categories and lower than the approval rates for general practioners and physiotherapists. Many claims can probably be prevented if chiropractors would prioritize informing patients about the normal course of their complaint and normal benign reactions to treatment.

## Background

The prevalence of adverse events (AE) following treatments is often overlooked when considering the overall effectiveness of an intervention. In spite of numerous high-quality trials published each day, the majority of these primarily focus on treatment effects and do not prioritize the reporting of AE [1]. Consequently, systematic reviews reporting on AE are generally lacking, accounting for only 10% of all reviews published [2]. The fact that many trials fail to report AE, may lead clinicians to believe that an intervention is harmless, when in reality its safety is unknown [3]. Accurate information on harms is imperative for evidence-based practice and as such AE should be reported from all parts of medicine [4]. Within musculoskeletal care, a plethora of AE have been published with regards to spine surgery [5], hip and knee surgery [6–9], corticosteroid injections [10,11], pharmacological therapy [12] exercise and physiotherapy [13–15] and manual therapy (MT) [16,17].

MT is an umbrella term covering a wide range of manual techniques and treatments including spinal manipulative therapy (SMT), mobilization and different soft-tissue techniques. Chiropractors, general practioners (GPs), physiotherapists and other manual therapists are among the health care professionals delivering MT. It is generally considered to be a safe and effective treatment option for patients with a range of musculoskeletal conditions [18]. However, a large number of mild AE have been reported and it is estimated that approximately 50% of patients will experience at least one adverse reaction during the course of treatment [16,17,19].

Particular controversy surrounds SMT [20,21], a MT procedure involving a high-velocity low amplitude thrust manoeuvre directed primarily towards the spine [22]. Earlier studies have shown that chiropractors are among practitioners who use SMT extensively, primarily for treatment of neck and low back pain [23]. The reports on adverse events following SMT range form mild, transitory discomfort [24,25] to serious, adverse events including stroke and death [26,27]. Some authors have advocated that there exists a massive under-reporting of serious injuries and that the risks associated with chiropractic treatment are much larger than previously thought [27]. In contrast, others have pointed to cases of misreporting in the medical literature, suggesting over-reporting of complications following chiropractic care, when clinicians are wrongly identified as chiropractors [28,29]. Furthermore, a recent study found that a large portion of AE commonly observed following chiropractic care may be the result of non-specific effects and natural history variation and not related to the treatment [30]. The concept of non-specific effects causing AE is not new [31], and this phenomenon has been observed in other clinical trials, where AE were noted following placebo treatments [32,33]. Clearly our current understanding of AE following MT is limited. Carnes et al. defined AE in manual therapies in 2010 through a modified Delphi consensus study [34] and concluded that reaching a consensus definition remains challenging.

The Danish Patient Compensation Association (DPCA) is the institution responsible for assessment of compensation claims following medical treatments performed by authorized health care personnel in Denmark. Since 2004, this has been the avenue for chiropractic patients and clinicians to report AE following chiropractic care if a financial compensation is considered relevant. In Norway, a similar system has existed since 2009 through the Norwegian System of Compensation to Patients (NSCP). An overview of registered claims to the two associations is available through their corresponding websites [35,36]. While reports on AE following MT are frequently published, our knowledge regarding the types of compensation claims reported from this field is limited. It should be noted, that while definitions of AE exist [34], compensation claims remain a different entity. Therefore, while it is impossible not to touch upon the field of AE, this paper is primarily concerned with compensation claims related to potential financial reimbursements after patient injury reports.

The purpose of this study was to report on the number and types of claims following consultation with chiropractors reported to the Danish and Norwegian compensation associations. Furthermore, we calculated age and gender distributions for the reported claims. Lastly, we assessed approval rates, appeals and financial compensations.

## Methods

### Study design

A retrospective study of compensation claims following consultations with chiropractors reported to the Danish and Norwegian compensation associations.

### Case selection

Eligible cases were registered with DPCA and NSCP between January 1st 2004 and December 31st 2012 and January 1st 2009 and December 31st 2012, respectively. Cases were included if they involved a chiropractor and they were finalized at the time of this review. Cases were excluded if the patient insurance law did not cover them, if patients withdrew claims, if claims were wrongly assigned to chiropractors, or if data were duplicates or missing.

### Data collection

The authors made direct contact with DPCA and NSCP independently. Microsoft Excel spread sheets were provided individually from the two associations containing data on each individual case during the time period. Spread sheets were provided in their raw form, and the first author individually assessed each case. When details were missing from the spread sheet, individual case files were manually accessed through a database and outstanding issues were resolved.

### Assessment of claims

Claims are assessed somewhat differently in Denmark and Norway, however both countries have a no fault system implying that the health care professional is not personally liable for accidents or injuries occurring in the office even in cases of negligence. In Denmark, cases are assessed based on criteria known as the *specialist standard* and the *rule of reason. Specialist standard* implies that a state-of-the-art procedure for the professional group in question was followed in every aspect of the consultation. If the clinician did not fullfill this standard, the patient likely receives compensation because the injury could potentially have been prevented if the clinician had adhered to this standard. The *rule of reason* implies that if a patient leaves the office of a health care professional in a worse condition than they entered under the given consultation complaint, they may receive compensation even in cases where the health care professional adhered to the *specialist standard*. This rule applies for instance in cases of cerebrovascular accidents occurring following cervical spine manipulation, where the primary complaint might be neck pain, even when no causal connection can be established. Thus, approved compensation for a claim does not imply causality or lack of judgement by the clinician but is a reflection of the statutory function of the compensation association.

In Norway certain criteria must be fulfilled in order to receive financial compensation: (1) There has to be a causal relationship between the treatment provided and the observed injury; (2) treatment should clearly not have been provided as there were clear signs of contraindication; (3) there has to be a financial loss because of the injury.

### Variables

Variables retained and included in this paper:

- Age
- Gender
- Registered date for claim and date for final decision
- Complaint categories
  - Worsening of symptoms – patients reported aggravated symptoms following treatment
  - Alleged disk herniation – patients claimed disk herniations had been induced because of treatment
  - Delayed referral – the chiropractor failed to refer the patient for adequate treatment
  - Newly developed symptoms/injury following treatment
  - Treatment induced fracture – patients reported treatment(s) resulting in bone fractures excluding cases concerning fractured ribs
  - Cervical artery dissection (CAD) – injuries related to either the internal carotid or the vertebral arteries
  - Rib injury (including fractures) to the rib(s) or associated cartilage
  - Accidents – minor unforeseen events such as patients falling off the treatment table
  - Miscellaneous such as alleged nerve and organ injuries
- Decisions (approved or rejected for financial compensation)
- Financial compensation (calculated in €EUR)
- Appeals
  - All claims can be appealed by the patient, either for approval if rejected or, if approved, for a higher financial compensation

Comparable claims were summarized and defined under the appropriate complaint category. Similar types of complaints constituting five or less cases were considered to be infrequent and categorized under miscellaneous.

### Analyses

First the annual complaint rate was calculated for Denmark and Norway individually based on registered time of claim. Then the study population was described with regard to age and gender using frequencies and proportions and for Denmark reported in relation to the annual distribution of patients in Danish chiropractic practice [37]. The overall annual frequency of claims and the annual approval rate was calculated for the two countries. Subsequently, complaints were categorized into complaint categories, which were tabulated and approval rates and financial compensations were calculated for each category. Lastly, claims were categorized based on the body region primarily treated and approval rates and financial compensations were reported based on these.

### Ethics

The spread sheets were anonymous and did not contain information that could identify the patient or chiropractor in question. In cases where details were missing and the database had to be accessed, sensitive information was available but was not withdrawn from the database or used in any way. The first author signed a confidentiality agreement before gaining access to the database.

## Results

**Figure 1:**
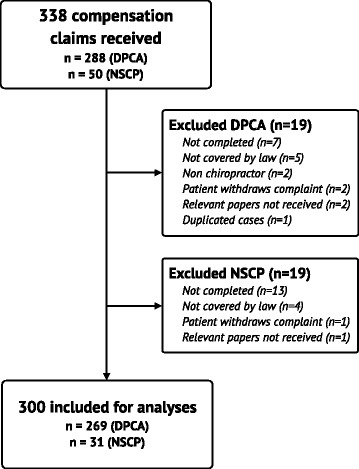
**Flowchart of the inclusion and exclusion process.**

From 2004–2012 a total of 338 claims were filed to DPCA (n = 288) and NSCP (n = 50). 38 cases were excluded for the following reasons: 20 were not completed at the time of review, 9 were not covered by the patient insurance law, 3 cases had incomplete data, in 3 cases the patient withdrew the claims, 2 cases involved non-chiropractors, and one case was a duplicate. Thus, a total of 300 claims were included in the analyses, 269 from DPCA and 31 from NSCP (Figure 1).

**Figure 2:**
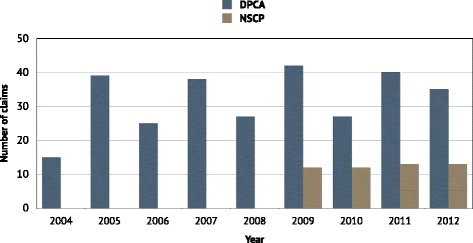
**Number of reported claims per year 2004 to 2012.** Number of annually reported claims to the two compensation associations since their respective inception.

**Figure 3:**
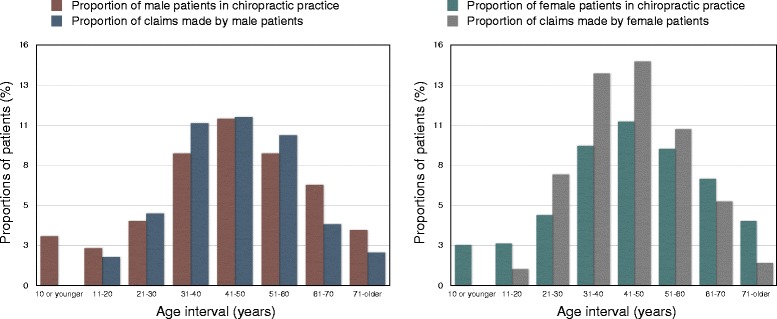
**Gender comparisons between chiropractic patients and reported compensation claims.** LEFT: Proportion (%) of male patients in Danish chiropractic practice in 2012 compared with proportion (%) of reported compensation claims by male patients in Denmark during 2004–2012. RIGHT: Proportion (%) of female patients in Danish chiropractic practice in 2012 compared with proportion (%) of reported compensation claims by female patients in Denmark during 2004–2012.

The annual rate of claims remained fairly stable during the period. The highest number observed in Denmark was in 2009 (n = 42) and in Norway in 2012 (n = 13) (Figure 2). Of the 300 included claims there were 166 (55.3%) females and 134 (44.7%) males. 217 patients were aged 31–60 years, accounting for 72.3% of the total study population. Statistics from 2012, suggests 57.9% of Danish chiropractic patients belong in this age interval [37]. The age distribution ranged from 11 to 80 years in the study population, in contrast to the annual patient population ranging from 0 to 99 years [37] (Figure 3).

Of 300 included claims, only 41 (13.7%) were eventually approved for financial compensation. Among the approved claims, 39 were from DPCA and the remaining two from NSCP. 32 of the 41 approved claims were directly approved following appraisal, while the remaining 9 were approved following appeals. A relatively stable approval rate was observed, ranging from its highest in 2004 (25%) to its lowest in 2010 (6.5%) (Table 1).

**Table 1:** **Overview of annual decisions**

	**2004**	**2005**	**2006**	**2007**	**2008**	**2009**	**2010**	**2011**	**2012**	**2013**	**Total**
DPCA											
Number of decisions	4	17	25	28	44	29	38	17	53	14	269
Approved/rejected	1/3	2/15	5/20	7/21	6/38	4/25	3/35	2/15	7/46	2/12	39/230
Approval percent	25%	11,8%	20%	25%	13,6%	13,8%	7,9%	11,8%	13,2%	14,3%	15.6%
Financial compensation (€EUR)	€240,630	€171,344	€630,468	€57,342	€555,566	€66,875	€20,848	€441,813	€43,457	€24,374	€2,252,717
NSCP											
Number of decisions						0	8	11	6	6	31
Approved/rejected						0	0/8	1/10	1/5	0/6	2/29
Approval percent						0	0	9,1	16,7	0	5.2%
Financial compensation (€EUR)						€0	€0	€1,440	€51,600	€0	€53,040
TOTAL											
Number of decisions	4	17	25	28	44	29	46	28	59	20	300
Approved/rejected	1/3	2/15	5/20	7/21	6/38	4/25	3/43	3/25	8/51	2/18	41/259
Approval percent	25,0	11,8	20,0	25,0	13,6	13,8	6,5	10,7	13,6	10,0	13.7%
Financial compensation (€EUR)	€240,630	€171,344	€630,468	€57,342	€555,566	€66,875	€20,848	€443,253	€95,057	€24,374	€2,305,757

Annual number of decisions, approvals and financial compensations in Denmark and Norway from 2004 to 2013.

The most frequent complaint categories were 91 (30.3%) cases of worsening symptoms following treatment, 57 (19%) cases of alleged disk herniations and 46 (15.3%) cases of delayed referral. Thus, these subgroups comprised 194 (65%) of the 300 claims. A total financial payment of €2,305,757 were distributed over the forty-one cases. The lowest compensation registered was €390 and the highest €605,480 (median €7,730, mean €56,238) (Table 2). Claims relating to treatment of specific body-regions included 93 (31%) claims after treatment to the cervical spine (31%), 48 (16%) to the thoracic spine and 118 (39.3%) to the lumbar spine (Table 3).

**Table 2:** **Overview of complaint categories**

**Complaint category**	**Total n (%)**	**Approved n (%)**	**Financial compensation €EUR (% of total)**
Worsening pain following treatment	91 (30.3%)	5 (5.5%)	€23,483 (1%)
Alleged disk herniation	57 (19%)	2 (3.5%)	€30,295 (1.3%)
Delayed referral	46 (15.3%)	14 (30.4%)	€148,667 (6.4%)
Newly developed symptoms/injury	35 (11.7%)	3 (8.6%)	€12,537 (0.5%)
Fracture	18 (6%)	3 (16.7%)	€20,612 (0.9%)
Cervical artery dissection	17 (5.7%)	11 (64.7%)	€2,044,523 (88.7%)
Rib injury	11 (3.7%)	0 (0%)	€0 (0%)
Accidents	10 (3.3%)	1 (10%)	€3,441 (0.1%)
Miscellaneous	15 (5.0%)	2 (13.3%)	€22,199 (1%)
TOTAL	300 (100%)	41 (13.7%)	€2,305,757

Complaint categories, approval rates and financial compensation based on total amount of claims from DPCA and NSCP.

**Table 3:** **Primary treated body region**

**Body region**	**Total n (%)/Approved n (%)**	**Financial compensation €EUR (% of total)**
Cervical spine	93 (31%)/18 (19.4%)	€2,107,271 (91.5%)
Thoracic spine	48 (16%)/4 (8.3%)	€18,236 (0.8%)
Lumbar Spine	118 (39.3%)/14 (11.9%)	€150,462 (6.5%)
Cervical and lumbar spine	3 (1%)/0 (0%)	€0 (0%)
Upper extremity	15 (5%)/2 (13.3%)	€9,359 (0.4%)
Lower extremity	10 (3.3%)/1 (10%)	€8,924 (0.4%)
Accidents	10 (3.3%)/1 (10%)	€3,441 (0.1%)
Non-specified	3 (1%)/1 (33.3%)	€8,064 (0.4%)
Total	300 (100%)/41 (13.7%)	€2,305,757

Complaints stratified by body region primary treated in relation to approvals and financial compensations based on total number of claims from DPCA and NSCP.

Out of the 300 included cases, a total of 84 appeals were registered. 71 (84.5%) decisions remained unchanged after appeal, 8 (61.5%) complaints that were originally rejected ended in favour of the patient and 5 (38.5%) in favour of the compensation associations. Of the 41 approved appeals, 32 (78%) were directly approved after appraisal. In one case (2.5%) an appeal for a higher compensation was filed but the payment remained unchanged but still approved for compensation for the original amount. The remaining 8 cases (19.5%) were approved after appeals.

## Discussion

This is the first study to provide a detailed description of compensation claims related to chiropractic treatment reported in Denmark and Norway. During 2004–2012 we were able to describe 300 of a total of 338 claims, only 41 of these were approved for compensation. A fairly stable rate of claims was observed, about thirty and twelve per year in Denmark and Norway, and the annual approval rate remained about 15% across the period.

Children under ten years of age regularly consult chiropractors [37,38] and Danish data suggests that 35% of patients in this age group are infants [37]. Paediatric treatment delivered by chiropractors remains a controversial topic within the medical literature [39]. Interestingly, our data did not reveal a single claim in this age group for both countries.

At the other end of the age spectrum, current data indicates that more than 20% of patients in chiropractic practice are over 60 years old [37]. In this study 37 claims were reported from this age group, representing 12.3% of the total amount. The United Nations predict that the proportion of people aged 60 years and over will triple over the next 40 years and will account for more than 20% of the world’s population by year 2050 [40]. Manifestations of musculoskeletal disorders increase with age [41] making interventions with documented effect and a favourable risk profile desirable [42]. In one recent study investigating the effectiveness of SMT and exercise interventions among seniors (aged 65 and above) with chronic neck pain [43] information on AE was systematically collected [44]. The AE identified were primarily musculoskeletal or pain related and non-serious and this is consistent with current evidence on AE following SMT and exercise [15,19,24,45] suggesting that these interventions have a lower risk of harm relative to other commonly used pharmacological interventions for neck pain [42].

Several types of compensation claims were reported following chiropractic treatment, however it appears that many complaints were filed because of unrealistic expectations to treatment effect or because the clinicians did not inform the patients about commonly occurring benign reactions to treatment. This is highlighted by the fact that three of the most frequent subgroups were “worsening symptoms”, “alleged disk herniation” and “newly developed symptoms” comprising 183 (61%) of the total complaints with only 10 (5.5%) being approved. While many patients seem to blame their symptoms on the treatment, it is probably due to the natural course of their underlying condition (i.e. degenerative joint disease) or non-specific effects, as supported by a recent randomized trial where the authors concluded that many of the normal AE seen following chiropractic care may be due to natural history variation and non-specific effects [30]. Therefore many complaints may be preventable if thorough and adequate information is given prior to delivering treatment.

Manual treatment of the cervical spine remains a controversial topic in health care [20,21]. In our data, 91 cases (31%) were based on complaints after manual treatment to the cervical spine and 18 were approved. A particular concern after cervical SMT is dissection of the vertebral and carotid arteries [46]. Seventeen claims concerning CAD were reported in this data, 14 in Denmark and three in Norway, and 11 of these were approved for financial compensation (64.7% approval rate) representing by far the highest approval rate across all complaint categories. All eleven approved claims were in Denmark, and all complaints were approved based on the *rule of reason* that does not imply causality. In Norway, however, all three cases were rejected because the clinician had displayed sound clinical judgement and because no contraindications for manual treatment were found. This illustrates the difference in the assessment of complaints between the two countries, as cases in Denmark are approved for compensation even though treatment is consistent with clinical guidelines and *specialist standard*. Interestingly, if CAD cases were excluded from this data the overall approval rate would decrease from 13.7% to 10.6% and the financial compensations would decrease by almost 90%. Furthermore, while the mean financial compensation was €56,238, the median payment was €7,730 illustrating that compensation for CAD to a large extent drives the cost of compensation at least in Denmark. Lastly, of the eleven approved CAD claims, five were reimbursed a total of €68,175, indicating that these were fairly mild with regards to affected workability and disability. The remaining six cases thus represented 97% of the financial compensation in this category.

Cases of delayed referral constitute the second most important subgroup with regards to approval rates with a total of forty-six claims and 14 (30.4%) approved cases. These claims consisted primarily of cases where the clinician had failed to act upon clear signs of nerve root compression, i.e. paresis and/or cauda equina syndrome, or did not refer for diagnostic imaging in spite of a clear indication. Furthermore, several cases in this category were approved because the clinician did not deliver adequate patient records resulting in approval in favour of the patient. In addition, many case files showed several examples of incomplete examinations and poor clinical judgment.

Norwegian (The Norwegian Health Economics Administration: Chiropractic services in Norway 2012, unpublished) and Danish chiropractors both deliver approximately two million consultations annually [37]. They receive on average 42 claims combined suggesting roughly one claim per 100.000 consultations. By comparison, Danish statistics show that in the period 2007–2012 chiropractors, GPs and physiotherapists (+ occupational therapists) received 1.76, 1.32 and 0.52 claims per 100.000 consultations, respectively [35,37] with approval rates of 13%, 25% and 21%, respectively. During this period these three groups were reimbursed on average €58,000, €29,000 and €18,000 per approved claim, respectively. Interestingly, while chiropractors generally seem to receive more claims per consultation than GPs and physiotherapists, the approval rate is substantially lower [35] and a similar trend is observed in Norway [36]. However, it is also evident that approved claims within chiropractic bear a higher financial burden than their peers. These numbers are clearly highly influenced by the cases related to CAD. Several reasons might explain a higher complaint rate within chiropractic but this remains speculation and we do not have hard evidence supporting any of the following suggestions: (1) chiropractic treatment might be perceived as more aggressive than that of GPs and physiotherapists (2) maybe scepticism towards chiropractic among medical physicians and physiotherapists could encourage more patient complaints (3) a higher out-of-pocket expense for chiropractic services compared with GP and physiotherapist services might influence the higher number of complaints (4) chiropractors do not adequately inform patients about normal side effects and reactions and patients regard these as serious and relevant for compensation claims (5) chiropractors encourage patients to report AE more frequently than GPs and physiotherapists.

### Strengths and limitations

The strengths of this study were that it included all filed compensation claims concerning chiropractic treatment reported to the two national compensation associations since their inception. As most studies related to MT primarily focus on treatment effects, this study provides important insights into what types of reactions chiropractic patients file as injuries to compensation bodies. One could expect the Norwegian numbers to increase in the coming years, as the registration system is still in its infancy whereas Danish data likely reflect the natural or “steady state” level within this particular system. Of importance, DPCA recently released its annual report for 2013, showing that complaints rates for GPs and physiotherapists have increased since 2011, while complaint rates for chiropractors have decreased. In addition, approval rates for chiropractors and physiotherapists have decreased in this period, while approval rates for GPs have remained stable [47].

Due to certain data missing and the small number of cases, a detailed analysis of chiropractor and treatment characteristics was not possible. Therefore we cannot assess whether differences in age, sex, educational background or years in practice influence the results and no pattern of injury reporting across these domains can be explored. Furthermore, no assessment around types of treatment and its relation to the AE reported was possible because exact description of the treatment was mostly absent. On the other hand one could argue that this reporting is pragmatic, as most chiropractors include several different interventions on most patients, including SMT, mobilization, exercise and different soft-tissue modalities [30]. Consequently, it would probably not be possible to discern which part of the treatment package was responsible for the compliant. Finally, because of the descriptive and retrospective nature of this paper, we cannot conclude on causality between the delivered treatment and the reported claims.

### Clinical and research implications

At this point it seems clear that a large portion of patients will experience minor AE during a course of chiropractic treatment [16,24,27,30]. For this reason chiropractors need to devote time to explain this to patients and emphasize that these are not AE but normal benign reactions to the manual treatment. While the causality between MT and CAD remains uncertain [20,21,48–50], these events will continue to occur in association with cervical spine manipulation. Evidence-based frameworks for early identification of CAD have recently been published [51] and we encourage clinicians to stay up-to-date on the evolving evidence surrounding CAD. As for cases of delayed referral, we can only recommend clinicians to stay vigilant and prioritize thorough and reasoned clinical examinations.

From a research perspective, it seems that chiropractors experience a higher rate of complaints following treatments when compared to physiotherapists and GPs. The reasons for this remain unknown, and thus qualitative research into these questions is of interest. Furthermore, we strongly support the implementation of incident reporting in chiropractic practice, and recently initiatives have been made in countries such as Switzerland and England. Although DPCA and NSCP are unique organizations in Europe, we suspect that more knowledge can be gained through more systematic collection of reported adverse events in chiropractic practice [52] as well as in medicine in general [3].

## Conclusion

The overall rate of compensation claims for chiropractors in Denmark and Norway is on par with GPs and physiotherapists and only a small proportion of claims result in compensation. Chiropractors in both countries receive more claims than GPs and physiotherapists but the approval rate is substantially lower. Many claims can probably be prevented if chiropractors would prioritize informing patients about the normal course of their complaint and normal benign reactions to treatment.
